# PDE-5 inhibitors should be used post radical prostatectomy as erection function rehabilitation? Opinion: No

**DOI:** 10.1590/S1677-5538.IBJU.2017.03.04

**Published:** 2017

**Authors:** Jonathan Clavell-Hernández, Run Wang

**Affiliations:** 1 Division of Urology, Department of Surgery, University of Texas Medical School at Houston, Houston, Texas, USA;; 2 University of Texas, MD Anderson Cancer Center, Houston, Texas, USA

**Keywords:** Phosphodiesterase 5 Inhibitors, Erectile Dysfunction, Penile Erection, Prostatectomy

## INTRODUCTION

The advancement and refinement in prostate cancer detection and treatment modalities have contributed to a younger patient population undergoing radical prostatectomy (RP) (1). Although it is effective in treating prostate cancer, radical prostatectomy has also been shown to compromise erectile function (EF), and therefore the patient’s quality of life and general well being (2). Alemozaffar et al. (3) attempted to predict erectile function after prostate cancer patients undergoing RP, external radiotherapy and brachytherapy. Pretreatment sexual health related quality of life score, age, serum prostate-specific antigen level, race/ethnicity, body mass index and intended treatment details were associated with functional erections 2 years after treatment. They found that 48% of patients (n=1027) with functional erections prior to treatment reported erectile dysfunction 2 years after treatment. In the prostatectomy cohort, 60% of patients with prior functional erections reported erectile dysfunction, along with 42% and 37% of the external radiotherapy and brachytherapy cohorts, respectively. The Prostate Cancer Outcomes study revealed 60% of men experienced self-reported erectile dysfunction 18 months after radical prostatectomy, and only 28% of men reported erections firm enough for intercourse at a 5-year follow-up (4). Many urologists believe more patients would be willing to undergo surgical treatment if it were not for the possibility of developing postoperative ED (2).

The discovery of the neurovascular bundle sparing technique by Dr. Patrick Walsh enabled urologists to provide hope of regaining erectile function after radical prostatectomy (5). However, despite meticulous dissection to preserve the neurovascular bundle, there is evidence that neuropraxia, ischemic and hypoxic nerve insults, fibrotic remodeling, and apoptosis of cavernous smooth muscle contribute to post-surgery erectile dysfunction (6).

After understanding the mechanisms that promote ED after radical prostatectomy, multiple studies have been focused on evaluating ways to increase oxygenation to the cavernosal bodies, decrease tissue fibrosis and apoptosis, and consequentially improve erectile function. In theory, the role of penile rehabilitation is to maintain tissue oxygenation and prevent tissue fibrosis until the cavernosal nerves recover from neuropraxia with the return of spontaneous nocturnal tumescence. However, evidence from our daily clinical practice demonstrates that penile rehabilitation does not necessarily guarantee the return of unassisted spontaneous erections.

By preventing the breakdown of cGMP, PDE-5 inhibitors may exert a protective effect on cavernosal smooth muscle after prostatectomy (7). However, despite their effectiveness in other forms of erectile dysfunction, their success in penile rehabilitation has not been proven to be as transparent. Padma-Nathan et al. (8) performed the first multicenter, double-blind, randomized, placebo-controlled trial to our knowledge investigating the effects of PDE5is on EF after RP. They randomized 125 patients into three treatment groups: placebo, Sildenafil citrate 50mg and Sildenafil citrate 100mg. Out of the 125 patients, only 76 completed the post-8-week washout evaluation period. After the post-washout period, only one of 25 patients (4%)in the placebo arm had adequate EF, versus 14 of 51 patients (27%) in the sildenafil 50mg and 100 mg groups combined (p=0.016). Although they suggested that nightly sildenafil has a benefit for patients with post-prostatectomy ED, there was a significant dropout rate which could call into question the statistical power of the study.

In 2008, Montorsi et al. (9) published a trial that investigated the effect of vardenafil in postoperative penile rehabilitation. This multicenter, double-blind placebo-controlled trial randomized 628 patients with a baseline International Index of Erectile Function (IIEF) score of >26 into taking nightly vardenafil, on-demand vardenafil, or placebo for 9 months. After 9-month treatment period, on-demand vardenafil was associated with more patients obtaining ≥22 on the EF domain of IIEF (IIEF-EF) score. However, after the 2 month washout period, there was no statistically significant difference in erectile function between groups. Similarly, dropout rates were substantial, ranging between 31%-35% in the study arms and there was no defined limit in the drug usage in the on-demand arm. Moreover, the data argued against the use of nightly PDE5i in the treatment of ED after radical prostatectomy.

Pavlovich et al. (10) pursued to investigate whether nightly sildenafil had an advantage over on-demand sildenafil. They randomized 100 men with good EF who had undergone nerve-sparing RP into two groups. The nightly sildenafil group consisted of patients taking nightly sildenafil and on-demand placebo; and the on-demand group consisted of on-demand sildenafil (with a maximum on-demand dose of 6 tablets per month) and nightly placebo starting the day after surgery for 12 months. All men had previously completed an IIEF-EF survey before surgery and had a score of ≥26 before undergoing nerve-sparing RP. Surgeons prospectively recorded the quality of NVB preservation, and this was quantified using a nerve sparing score (NSS) of one to four, with higher scores representing better preservation. The double-blind study period included quality of life assessments every 3 months for 12 months after RP, and a final assessment at 13 months after a washout period of 1 month. Compliance in returning questionnaires ranged from 60%-96% per time-point but was balanced between groups. After adjusting for potential confounding factors, no significant differences were found in EF between treatments at any single time-point after RP. NSS was the only factor that was consistently found to have a significant association with EF outcomes in all longitudinal multivariable models. This study did show some limitations. First, fearing that patients would not want to be randomized to a placebo-only group, a pure placebo arm was not part of the trial. Moreover, 90% of subjects were Caucasian which is not generalizable to all populations.

The REACTT study conducted by Montorsi et al. (11) aimed to compare the efficacy of tadalafil daily and on demand versus placebo in improving unassisted EF and reducing loss of penile length following nerve-sparing RP. Four hundred twenty-three patients were randomized into 9 months of treatment with tadalafil 5mg once daily, tadalafil 20mg on demand, or placebo followed by a 6-week washout period and 3 months open-label tadalafil once daily (to all patients). At 9 months, they found a significant difference in reaching target IIEF-EF ≥22 in the tadalafil once daily group compared to placebo. However, after the drug free washout period, there was no significant difference in EF between groups. After the open-label tadalafil once daily period, IIEF-EF scores increased in all treatment groups. Regarding penile length, there was significant protection from penile length loss in the daily tadalafil group (2.2mm) compared to other groups (7.9 mm on demand, 6.3mm placebo) at 9 months of treatment. Mulhall et al. reported a descriptive post-hoc analysis using a more strict definition for EF-recovery of returning back to the pre-surgery IIEF-EF level (“back-to-baseline analysis”). However, this had no major impact on results and showed no effect on unassisted EF following treatment cessation after 9 months (12).

All these studies evaluated the use of PDE5is by relying on self-reported outcomes to determine efficacy of therapy which could lead to response bias. Kim et al. (13) conducted a study to evaluate the effects of nightly sildenafil therapy using a more objective approach with nocturnal penile rigidity (RigiScan TM, Gotop Medical, Inc., St Paul, MN, USA) in addition to the IIEF-EF score. They randomized 97 patients of which 74 completed the study into taking daily sildenafil with on-demand sildenafil or daily placebo with on-demand sildenafil. Outcomes were evaluated every 3 months for 12 months and at 13 months after 1 month wash-out period. They noted no significant difference in EF between treatment groups based on IIEF-EF domain or RigiScan, suggesting that nightly sildenafil has no benefit over on-demand sildenafil.

## CONCLUSIONS

Although there is not enough evidence to create an algorithm for penile rehabilitation, the use of PDE5-inhibitors has been well-tolerated and no significant harm of rehabilitation has been demonstrated provided the patients understand the side-effects and costs. This has driven urologists to include penile rehabilitation programs in their practices (14). Most have adopted the use of PDE5-inhibitors, either as monotherapy or in combination with other modalities. However, if we base our practice according to current data, the possibility exists that many urologists have integrated penile rehabilitation with PDE5-inhibitors into their practices based more on theoretical hope than concrete evidence. We have noted that research in penile rehabilitation is leading towards the use of combination therapies (15). The ideal rehabilitation modality should consist of one that is effective, convenient, not too expensive and exerts minimal side effects on the patient. Current research lacks convincing data that PDE5 inhibitors contribute to the complete restoration of spontaneous erectile function.
